# Trends in Alzheimer's‐Related Mortality Among Type 2 Diabetes Patients in the United States: 1999–2019

**DOI:** 10.1002/edm2.70032

**Published:** 2025-02-03

**Authors:** Saad Ahmed Waqas, Dua Ali, Taimor Mohammed Khan, Shaheer Qureshi, Hibah Siddiqui, Maryam Sajid, Zahra Imran, Hussain Salim, Muhammad Umer Sohail, Raheel Ahmed, Shayan Marsia

**Affiliations:** ^1^ Department of Medicine Dow University of Health Sciences Karachi Pakistan; ^2^ National Heart and Lung Institute Imperial College London London UK; ^3^ Department of Neurosciences Corewell Health/Michigan State University Grand Rapids Michigan USA

**Keywords:** Alzheimer's disease, health disparities, mortality, trends, type 2 diabetes mellitus

## Abstract

**Background:**

Recent research has shown that type 2 diabetes mellitus (T2DM) has increased the burden of Alzheimer's disease (AD) in the US aging population. However, trends in mortality from this comorbidity among adults aged ≥ 65 years have not been investigated.

**Objectives:**

This study examined trends and disparities in AD‐related mortality among older US adults with T2DM from 1999 to 2019.

**Methods:**

Data from the CDC WONDER database were analysed to assess AD‐related mortality in patients with T2DM aged ≥ 65 between 1999 and 2019. Age‐adjusted mortality rates (AAMRs) per 100,000 people and annual percent change (APC) were calculated and stratified by year, sex, race/ethnicity, age, urbanisation and geographical region.

**Results:**

From 1999 to 2019, there were 71,550 deaths with T2DM and AD among adults aged ≥ 65. AAMRs rose from 4.12 in 1999 to 11.65 in 2019, with the sharpest increase between 2014 and 2017 (APC: 10.81; 95% CI: −3.20 to 13.43). Women had slightly higher AAMRs than men, with rates increasing from 4.71 in 1999 to 11.61 in 2019 for women, and from 4.08 to 11.70 for men. Hispanic individuals saw the highest increase in AAMR (11.15), followed by non‐Hispanic Black (9.30) and White populations (7.92). AAMRs were highest in the West (10.91) and the Midwest (9.62), while the Northeast (4.70) had the lowest. Nonmetropolitan areas had consistently higher AAMRs (10.74) than large metropolitan areas (6.68) and small/medium metropolitan areas (9.25). States in the top 90th percentile for T2DM–AD mortality included California, South Dakota and Kentucky, where rates were approximately eight times higher than in states in the lowest 10th percentile.

**Conclusions:**

This study reveals a significant rise in T2DM–AD comorbidity‐related mortality among older adults, especially among Hispanics, women and rural residents. These findings underscore the need for targeted interventions to reduce the burden in vulnerable populations.

## Graphical abstract

Alzheimer's‐related deaths among individuals with type 2 diabetes among older adults in the United States increased significantly from 1999 to 2019, with notable racial, geographical and rural–urban disparities. Hispanics and rural residents experienced the highest age‐adjusted mortality rates, emphasising the need for targeted interventions to address these inequities in Alzheimer's care within diabetic populations.

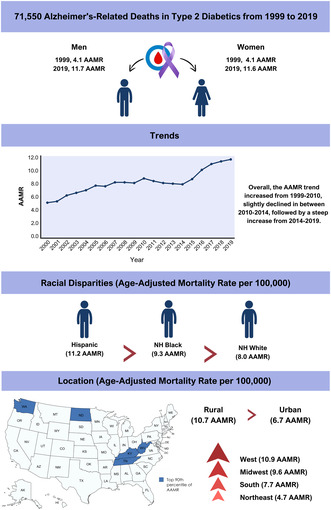

**SUMMARY**

- Impact statement
  ○ We certify that this work is confirmatory of recent novel clinical research.
  ○ It is consistent with previous literature such as: https://doi.org/10.3389/fnins.2018.00383; https://doi.org/10.3390/biomedicines10040778; https://doi.org/10.1111/jnc.15166; https://doi.org/10.1111/joim.12534.
  ○ This study uniquely examines trends in Alzheimer's disease (AD)‐related mortality in type 2 diabetes mellitus (T2DM) patients, highlighting demographic and geographical disparities not previously explored. Our findings reveal increased AD‐related mortality among elderly Hispanic individuals, rural populations and women with T2DM, emphasising the need for targeted public health interventions for these vulnerable groups.
- Key points
  ○ The age‐adjusted mortality rate for AD among older adults with T2DM increased significantly from 1999 to 2019.
  ○ Hispanic individuals, women and rural residents have the highest mortality rates from T2DM–AD comorbidity.
  ○ States with the highest mortality rates include California, South Dakota and Kentucky, emphasising regional disparities in disease burden.
- Why does this matter?
  ○ These findings reveal a critical need for targeted interventions to address the disproportionate mortality burden of T2DM and AD comorbidity among vulnerable populations.
  ○ Reducing disparities in these high‐risk groups could improve health outcomes for older adults and reduce healthcare costs associated with these conditions.

## 1 Introduction

Globally, the incidence of type 2 diabetes mellitus (T2DM) is on the rise, reaching epidemic proportions and afflicting over 400 million people [1]. One in ten Americans is said to have T2DM [2]. In the United States, Alzheimer's disease (AD) is the fifth leading cause of mortality in people aged 65 and older [3]. The estimated total healthcare costs for the treatment of Alzheimer's in 2024 are projected to be $360 billion, excluding the portion of unpaid caregiving in the US [4]. Diabetes is one of the four major non‐communicable diseases [5], and recent research has pointed to a concerning trend: Alzheimer's‐related mortality among patients with T2DM appears to be rapidly increasing [6].

In the last decade, there has been a surge in the number of cases of T2DM and AD. A meta‐analysis revealed that patients with T2DM have a 73% increased risk of developing all‐type dementia and a 56% increased risk of AD [7]. Additionally, previous research reports that up to 80% of people with AD also have T2DM [8, 9]. A longitudinal cohort study further highlighted that diabetics have a 65% higher risk of developing AD than non‐diabetics [10]. Several studies show that T2DM and AD have several common pathophysiological factors [11, 12, 13]. The connection is so pronounced that AD is often referred to as ‘type 3 diabetes’ [14]. This form is characterised by insulin resistance in the brain, leading to disruptions in central insulin signalling processes, neuronal dysfunction and neurodegeneration [15, 16].

By 2060, the number of AD cases in the United States is predicted to triple [17]. Therefore, corroborating the effect of T2DM on the brain and neurodegenerative diseases is imperative because of the evidence provided above and the rapidly increasing rates of T2DM [18]. A sedentary lifestyle, obesity and complex inheritance–environment interactions are risk factors for T2DM [19]. The present study aimed to describe racial differences in AD‐related mortality in T2DM patients in the US population aged 65 and above, between 1999 and 2019, taking into account temporal trends and variation by sex, geographical areas and urban–rural distinctions. Examining these trends and disparities in Alzheimer's‐related mortality among patients with T2DM is of great significance, as it has the potential to reveal underlying mechanisms, inform targeted interventions and promote health equity for minorities.

## 2 Methodology

### 2.1 Population and Study Setting

We conducted a comprehensive study utilising the Centers for Disease Control and Prevention's WONDER (Wide‐Ranging Online Data for Epidemiologic Research) database to retrieve information from death certificates to assess Alzheimer's mortality secondary to T2DM among individuals aged 65 or older during the period 1999–2019. Our study focuses on this age group as AD is most prevalent among adults older than 65, with the risk of the condition increasing significantly with age [20]. The study utilised codes from the International Statistical Classification of Diseases and Related Health Problems, 10th Revision (ICD‐10). We selected individuals with both AD (*ICD‐10* code: G30) [21] and T2DM (*ICD‐10* code: E11) [22] recorded as contributing or underlying causes of death. As a comparison cohort, all deaths recorded with AD alone as a contributing cause of death among adults aged 65 or older were included. The research did not require approval from a regional institutional review board because it was based on de‐identified public use data issued by the government. This study adhered to STROBE standards for reporting observational research.

### 2.2 Data Abstraction

The data included age, sex, race or ethnicity, and location of death, which were organised according to demographic factors. People died in various locations, including outpatient, emergency room, inpatient, death on arrival or status unknown, home, hospice and nursing home/long‐term care facility. Race and ethnic groups were classified into two types: Hispanics (Latino) and non‐Hispanics (NH), which included NH White individuals and NH Black or African American individuals. NH American Indian or Alaska Native and NH Asian or Pacific Islander populations were not included as data for these groups were unavailable for multiple years. Age groups were classified as 65–74, 75–84 and 85+ years of age. We classified the population geographically into urban and rural regions using the National Center for Health Statistics Urban–Rural Classification Scheme. Urban areas included large metropolitan regions with populations exceeding 1 million and small to medium metropolitan regions with populations ranging between 50,000 and 1 million. Rural areas, in contrast, were defined as regions with populations under 50,000, along with certain additional counties as specified by the 2013 US Census. We divided the United States into four regions—Northeast, Midwest, South and West—according to the classification by the US Census Bureau [23].

### 2.3 Statistical Analysis

Age‐adjusted mortality rates (AAMRs) and crude mortality rates per 100,000 were extracted from the CDC WONDER database. To examine changes in mortality patterns related to Alzheimer's secondary to T2DM, we calculated the percentage change in AAMR per 100,000 individuals from 1999 to 2019 using the 2000 US population as a baseline for AAMR standardisation [24]. AAMR was used for all analyses except those stratified by age categories, where the crude mortality rate was used. These rates were classified into categories by year, sex, race/ethnicity, urbanisation and census data, with a 95% confidence interval (CI).

We used the Joinpoint Regression Program (Joinpoint V 5.2.0, National Cancer Institute) [25] to analyse variations in the mortality rate over time. Through this, we calculated the annual percent change (APC) and its associated 95% CI in the AAMRs. This method involved fitting log‐regression models to identify significant changes in AAMR where temporal variation occurred. Two‐tailed *t*‐testing evaluated the APCs as increasing or decreasing if the slope reflecting changes in mortality significantly deviated from zero. We also evaluated the average annual percentage change (AAPC) and its associated 95% CI through the Joinpoint Regression Program, which is calculated by fitting a regression line to the natural logarithm of rates. Lastly, a test for parallelism was performed to evaluate the significance of the differences in the trends between the two cohorts (T2DM‐ and AD‐related mortality vs. all AD‐related mortality) stratified by various factors (such as gender, race, age and urbanisation categories). The significance level of *p* < 0.05 was considered statistically significant.

## 3 Results

Between 1999 and 2019, 71,550 deaths from AD were recorded among older adults (aged ≥ 65 years) with type 2 diabetes (Table S1). Across every stratification, T2DM patients experienced a significantly higher rise in AD mortality trends than the overall population (Table 1). Most of these deaths (57.4%) occurred in nursing homes or long‐term care facilities, followed by deaths at the decedent's home (20.8%) and medical facilities (13.7%). A small number of cases (0.2%) had an unknown place of death, while another 0.2% were declared dead upon arrival at a medical facility (Table S2).

**TABLE 1:** Age‐adjusted mortality rate (AAMR) and average annual percent change (AAPC) of AD‐related mortality in the overall population and in T2DM patients.

	Overall population (per 100,000 population)			Population with T2DM as a contributing cause of mortality (per 100,000 population)			AAPC 1999–2019 (95% CI) for overall population		AAPC 1999–2019 (95% CI) for T2DM patients		*p* value for AAPC in T2DM versus overall population
	1999	2019	Percent change 1999–2019	1999	2019	Percent change 1999–2019	AAPC	(95% CI)	AAPC	(95% CI)	
Overall	228	281.9	23.64%	4.1	11.7	185.40%	1	(0.7, 1.3)	5	(4.5, 5.8)	0.002
Sex											
Male	211.1	232.3	10%	4.1	11.7	185.40%	1.4	(0.99, 1.69)	5.1	(4.5, 6.2)	< 0.001
Female	243.4	232.4	−4.50%	4.1	11.6	182.90%	0.5	(0.3, 0.8)	5.5	(5.1, 6.2)	0.002
Age^a^											
65–74	30.2	30	−0.66%	0.6	1.3	116.70%	−0.09	(−0.4, 0.27)	2	(1.0, 3.2)	0.005
75–84	231.8	255.9	10.40%	5.1	12.6	147.10%	0.5	(0.2, 0.7)	4.6	(4.2, 5.1)	0.002
85+	1059	1429.9	35%	16.3	53	2252%	1.4	(1.1, 1.8)	5.8	(5.3, 6.5)	0.002
Race											
NH white	238.5	296.2	24.20%	4.1	11	168.30%	1.03	(0.7, 1.3)	4.8	(4.2, 5.5)	0.006
NH black or African American	182.1	259.3	42.40%	5	12.3	146%	1.7	(1.5, 1.9)	4.9	(4.3, 5.8)	< 0.001
Hispanic or Latino	138.8	241.9	74.30%	2.9	17.4	500%	2.9	(2.5, 3.5)	6.7	(6.0, 7.8)	< 0.001
Region											
Northeast	184.3	181.3	−1.60%	3.1	6	93.50%	−0.2	(−0.5, 0.09)	3.2	(2.7, 4.0)	0.982
Midwest	237.3	296.8	25.10%	5	13.4	168%	1.1	(0.9, 1.4)	4.7	(3.9, 5.9)	0.999
South	231.4	294.8	27.40%	4.6	10.1	119.60%	1.1	(0.8, 1.4)	4.1	(3.6, 4.8)	0.993
West	259.1	333.7	28.80%	3.3	17.4	427.30%	1.3	(1.0, 1.6)	7.9	(7.2, 9.2)	0.562
Urbanisation											
Large metropolitan	213.3	256.7	20.40%	3.1	10.2	229%	0.9	(0.7, 1.1)	5.7	(5.4, 6.1)	< 0.001
Small/medium metropolitan	243.8	305.6	25.30%	4.9	12.7	159.20%	1	(0.7, 1.3)	5.1	(4.5, 5.9)	0.002
Non‐metropolitan	240.5	314.3	30.70%	5.4	14.3	164.80%	1.2	(1.0, 1.6)	4.5	(3.7, 5.5)	0.012

*Note:* *p* < 0.05 signifies that the trends in AD‐related mortality are significantly different in T2DM patients than in the overall population.

^a^ Crude rates were used instead of AAMRs.

### 3.1 Annual Trends for AD and T2DM‐Related Mortality

**FIGURE 1:**
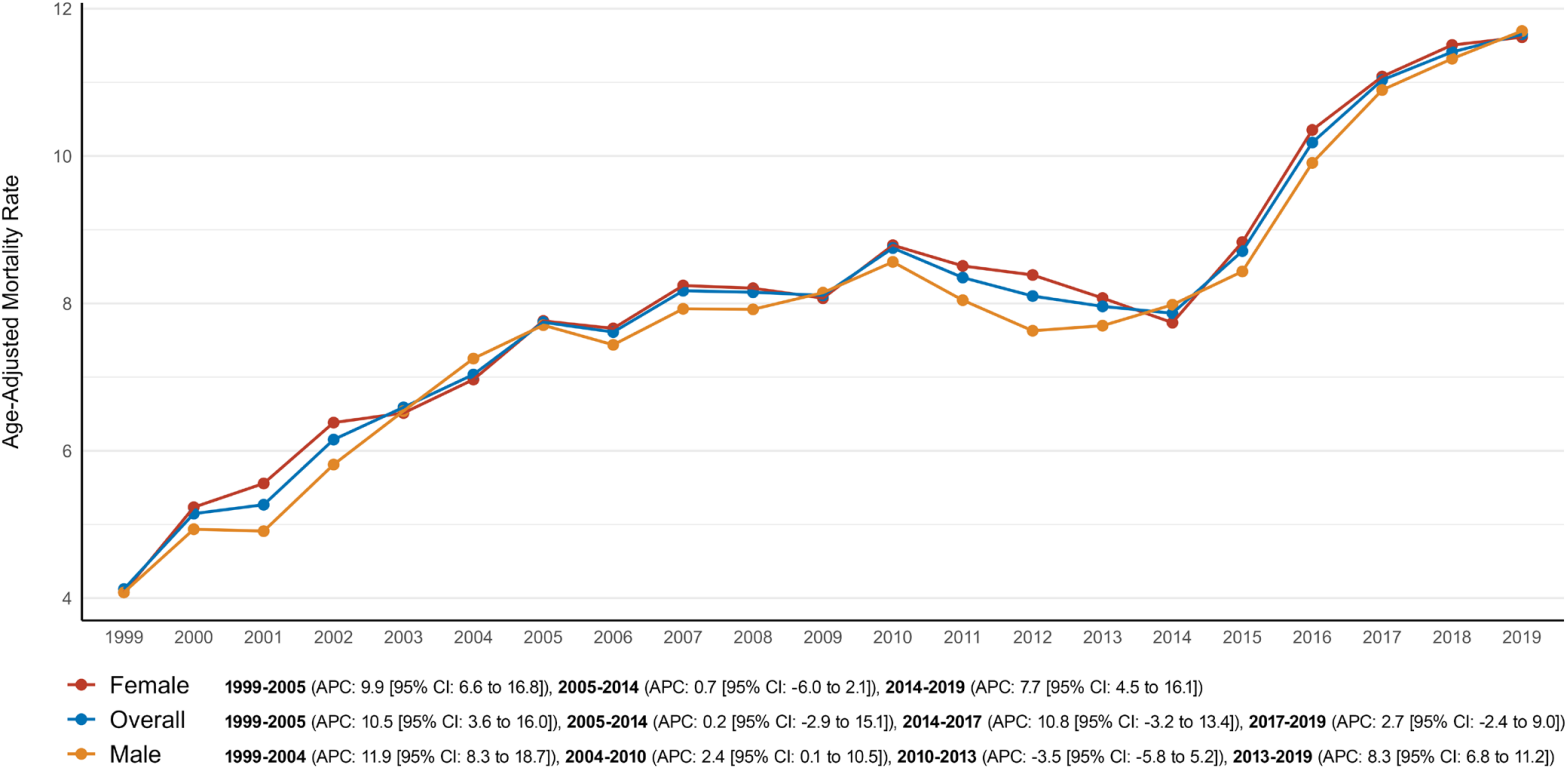
Trends in overall and sex‐stratified age‐adjusted AD and T2DM‐related mortality per 100,000 in the United States from 1999 to 2019.

The AAMR for AD and T2DM comorbid deaths in adults over 65 increased from 4.1 in 1999 to 11.7 in 2019. The overall AAMR saw a sharp rise between 1999 and 2005, with an annual percentage change (APC) of 10.5% (95% CI: 3.6%–16.0%). From 2005 to 2014, this increase slowed significantly, with an APC of 0.2% (95% CI: −2.9% to 15.1%). However, between 2014 and 2017, the rate spiked again, showing an APC of 10.8% (95% CI: −3.2% to 13.4%). Afterwards, the AAMR continued to rise, maintaining an upward trajectory until the study's end, with an APC of 2.7% (95% CI: −2.4% to 9.0%) (Figure 1, Table S3).

### 3.2 AD and T2DM‐Related Mortality Stratified by Demographics

#### 3.2.1 Gender‐Wise Analysis

Throughout the study period from 1999 to 2019, female AAMR remained slightly higher than male AAMR (overall AAMR for women: 8.3 [95% CI: 8.2–8.3]; for men: 8.2 [95% CI: 8.1–8.3]). For women, the AAMR rose from 4.1 in 1999 to 7.8 in 2005 (APC: 9.9% [95% CI: 6.6%–16.8%]), followed by a modest increase to 8.5 in 2014 (APC: 0.7% [95% CI: −6.0% to 2.1%]). By the end of the study period, the AAMR reached 11.6 (APC: 7.7% [95% CI: 4.5%–16.1%]). For men, the AAMR similarly increased, starting at 4.1 in 1999 and rising to 7.3 in 2004 (APC: 11.9% [95% CI: 8.3%–18.7%]). This was followed by a gradual incline until 2010 (APC: 2.4% [95% CI: 0.1%–10.5%]), a slight decline until 2013 (APC: −3.5% [95% CI: −5.8% to 5.2%]) and then a final rise to 11.7 by 2019 (APC: 8.3% [95% CI: 6.8%–11.2%]) (Figure 1; Table S3).

#### 3.2.2 Race‐Wise Analysis

The AAMR for all ethnicities showed significant increases over the study period, with Hispanic or Latino people experiencing the largest rise (overall AAMR: 11.2 [95% CI: 10.7–11.4]), followed by NH Black or African American populations (overall AAMR: 9.3 [95% CI: 9.1–9.5]) and NH White populations (overall AAMR: 8.0 [95% CI: 7.9–8.1]) (Table S5).

For NH Black or African Americans, the AAMR rose from 5.0 in 1999 to 9.2 in 2006 (APC: 11.2% [95% CI: 8.4%–14.8%]). This trend then steadily declined until 2014 (APC: −1.1% [95% CI: −4.3% to 3.5%]), followed by a steep increase until 2017 (APC: 12.0% [95% CI: −0.5% to 15.5%]) and a slight decline to 2019 (APC: −3.1% [95% CI: −8.4% to 5.1%]). NH Whites saw a significant increase in AAMR from 1999 to 2005 (APC: 10.1% [95% CI: 7.1%–15.7%]), after which the rate remained stable until 2014 (APC: −0.1% [95% CI: −5.7% to 1.3%]). A subsequent rise was observed until the end of the study period (APC: 7.9% [95% CI: 4.8%–16.1%]). In contrast, Hispanic or Latino AAMR showed a substantial increase throughout the study (APC: 6.7% [95% CI: 6.0%–7.8%]) (Figure S1).

#### 3.2.3 Age‐Wise Analysis

The overall crude rates for the 65–74 year, 75–84 year and 85+ year age groups were 1.1 (95% CI: 1.0–1.1), 9.1 (95% CI: 9.0–9.2) and 36.1 (95% CI: 35.8–36.5), respectively (Table 1; Table S4).

The 85+ year age group exhibited a fluctuating trend with an AAPC of 5.8% (95% CI: 5.3%–6.5%), compared to an AAPC of 4.6% (95% CI: 4.2%–5.1%) for the 75–84 year age group. Contrastingly, the 65–74 year age group showed a steady rise throughout the study period with an AAPC of 2.0% (95% CI: 1.0%–3.2%) (Table 1; Figure S2).

### 3.3 AD and T2DM‐Related Mortality Stratified by Region

#### 3.3.1 Urbanisation‐Wise Analysis

During the study period, non‐metropolitan areas consistently showed higher AAMRs for AD and T2DM comorbidity (AAMR: 14.3 [95% CI: 13.5–15.1]) than small/medium metropolitan areas (AAMR: 9.3 [95% CI: 9.1–9.4]) and large metropolitan areas (AAMR: 6.7 [95% CI: 6.6–6.8]) (Table S6).

Both large metropolitan and non‐metropolitan areas experienced a sharp rise in AAMRs from 1999 to 2006, with APCs of 8.9% (95% CI: 7.1%–10.9%) and 9.9% (95% CI: 6.9%–15.1%), respectively. Afterwards, the AAMR in large metropolitan areas increased more steadily until 2014 (APC: 1.6% [95% CI: 0.1%–7.2%]), while non‐metropolitan areas saw a slight decline during the same period (APC: −1.8% [95% CI: −9.4% to 0.2%]). However, non‐metropolitan AAMR then surged sharply until the end of the study (APC: 7.4% [95% CI: 3.4%–17.8%]). Similarly, large metropolitan areas saw a steep rise in AAMR until 2017 (APC: 11.3% [95% CI: 1.3%–13.2%]), followed by a modest increase for the remainder of the study (APC: 3.2% [95% CI: 0.2%–7.9%]).

**FIGURE 2:**
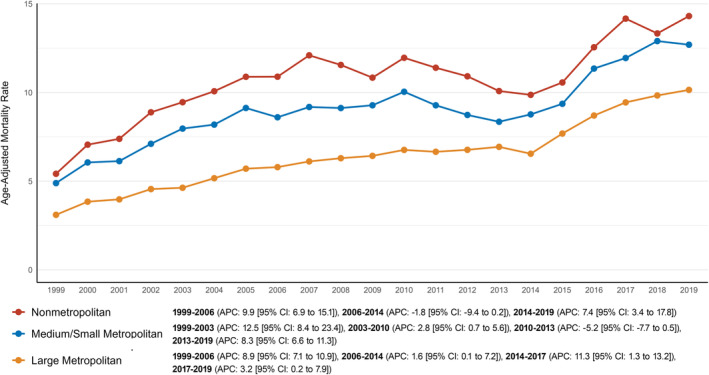
Trends in AD and T2DM‐related mortality per 100,000 stratified by the level of urbanisation in the United States from 1999 to 2019.

Small/medium metropolitan areas exhibited a more complex trend. Initially, there was a sharp increase in AAMR until 2003 (APC: 12.5% [95% CI: 8.4%–23.4%]), followed by a slight rise through 2010 (APC: −5.2% [95% CI: −7.7% to 0.5%]). This was followed by a decline until 2013 (APC: −5.2% [95% CI: −7.7% to 0.5%]), after which the AAMR saw a sharp rise again until the study's conclusion (APC: 8.3% [95% CI: 6.6%–11.3%]) (Figure 2).

### 3.4 State and Census Region‐Wise Analysis

**FIGURE 3:**
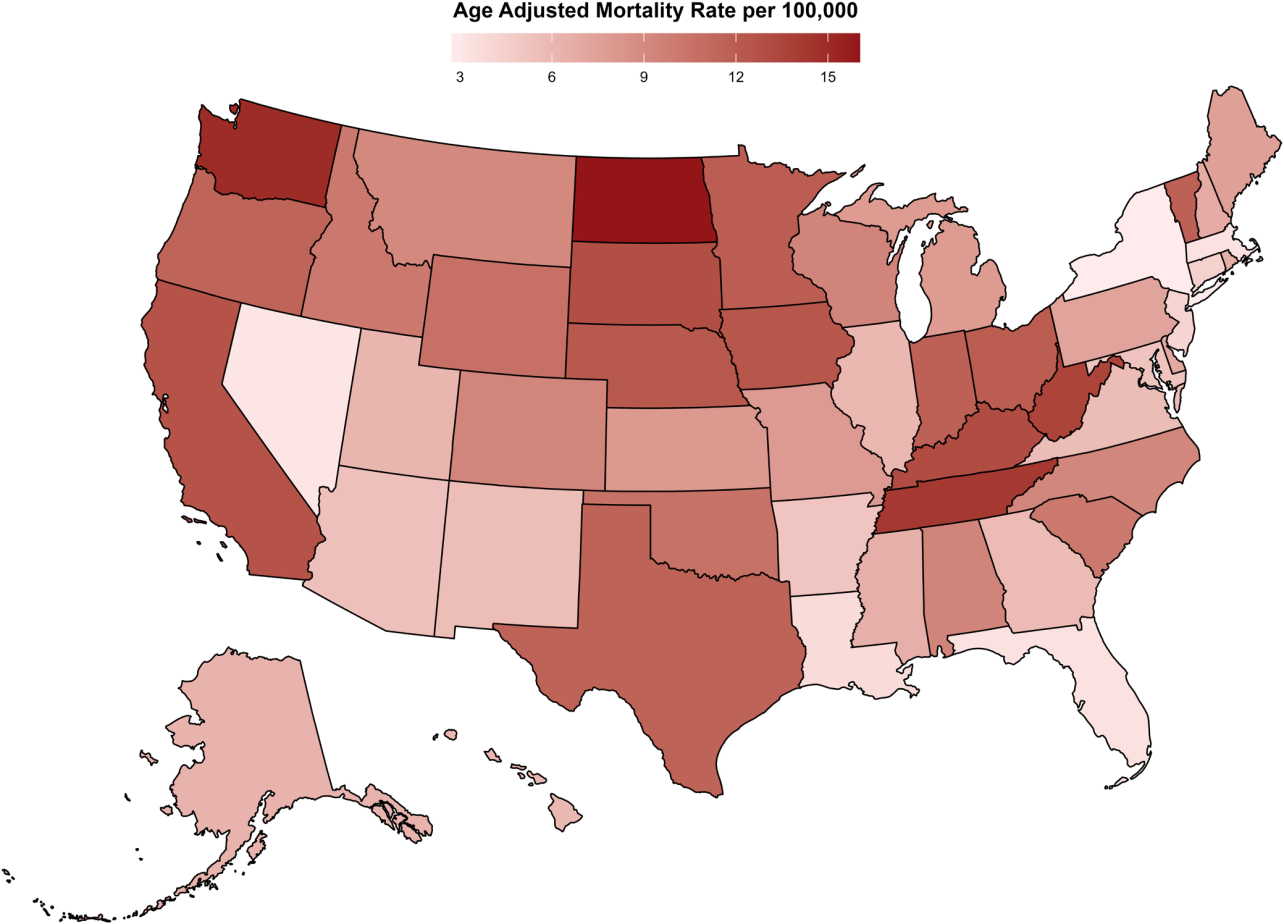
AD and T2DM‐related mortality per 100,000 stratified by state in the United States from 1999 to 2019.

A significant disparity in AAMR was evident across states, with values ranging from 2.7 in New York (95% CI: 2.6–2.9) to 16.0 in North Dakota (95% CI: 14.4–17.6) (Table S8). States in the top 90th percentile, including California, South Dakota, Kentucky, West Virginia, Tennessee, Washington and North Dakota, showed an approximately eightfold increase in AAMRs compared to those in the lower 10th percentile—New Jersey, Louisiana, Florida, Massachusetts, Nevada and New York (Figure 3; Figure S4).

Throughout the study, the Western region recorded the highest mortality rates (overall AAMR: 10.9 [95% CI: 10.8–11.1]), followed by the Midwestern region (overall AAMR: 9.6 [95% CI: 9.5–9.8]), Southern region (overall AAMR: 7.8 [95% CI: 7.7–7.9]) and Northeastern region (overall AAMR: 4.7 [95% CI: 4.6–4.8]) (Figure S3, Table S7).

## 4 Discussion

Diabetes is a complex global disease associated with other chronic complications such as cardiovascular diseases and kidney failure. Recent studies have highlighted a link between type T2DM and AD, though few have examined the demographic disparities in mortality [26, 27]. Using national death certificate data, we observed several important findings. First, a significant increasing trend was identified in mortality from AD in older adults with T2DM, with almost a threefold increase in AAMR between 1999 and 2019. Second, women had a higher AAMR than men during the study period. Third, Hispanics had the highest Alzheimer‐related T2DM mortality among all the racial and ethnic groups in the US. Fourth, rural areas had consistently higher mortality trends than urban areas. Further, AAMR varied geographically, with states in the upper 90th percentile of AD and T2DM‐related mortality having almost eight times higher mortality than those in the lower 10th percentile. The overall AD population experienced a significantly smaller increase in AAMR across all variables than those with T2DM as a contributing factor. These findings underscore the rising burden of diabetes in the US population and reinforce the role of T2DM as a significant risk factor for AD, as suggested by previous research [21, 27, 28]. Notably, this trend exhibited a greater incline than the overall mortality trend of AD within the same timeframe [29].

Emerging evidence strongly suggests a pathophysiological link between T2DM and AD. Neuropathological changes in T2DM, such as brain atrophy, reduced glucose metabolism due to insulin resistance and neuronal loss, closely mirror those seen in AD. Impaired glucose transport to the brain compromises neuronal energy, contributing to synaptic dysfunction and cognitive decline. These changes indicate that T2DM accelerates AD pathology and directly contributes to cognitive impairment, independent of traditional AD risk factors [30, 31]. Key mechanisms linking T2DM and AD include hyperglycaemia, which damages cerebral blood vessels and impairs blood flow, and insulin resistance, which promotes tau phosphorylation, a hallmark of AD [32]. Additionally, hyperinsulinemia may increase amyloid‐beta levels by competing for degradation, further contributing to AD pathology [33]. Early intervention in T2DM, through lifestyle changes and glucose‐lowering therapies, could reduce AD risk.

An important observation from our data resulted from comparing sex differences. Both genders exhibited an increased AD and T2DM‐related mortality rate, but women marginally had a higher AAMR than men. This finding is unexpected, given that females demonstrated a decline in overall Alzheimer's‐related mortality, and as previous literature indicates, men are generally diagnosed with T2DM more commonly [34]. This adverse health outcome in women may be due to their higher burden of risk factors. For instance, psychological stress has been linked to an increased risk of AD [35]. Women who experience sustained high stress are more likely to develop AD than men [35]. Additionally, brain differences between men and women may contribute to biological differences in T2DM‐related pathways, such as the accumulation of β‐amyloid (Aβ) plaques, which are found more often in women [36]. Espeland et al. reported that women with T2DM who were assigned hormone therapy at 65 years or more had a substantially increased risk for lower brain volumes than those without T2DM, suggesting that oestrogen‐based therapies may further accentuate the risk for brain atrophy, specifically of grey matter, in older women. Such results were not found in men [37]. Women's longer life expectancy may also explain their higher incidence of AD compared to men [38, 39]. These findings highlight the need for targeted prevention strategies to address sex‐specific risk factors in women with T2DM to reduce their higher risk of Alzheimer's.

Our analysis reveals that individuals of Hispanic background have higher age‐adjusted Alzheimer's‐related T2DM mortality rates than other racial groups over the last two decades, likely due to the larger population burden of T2DM among Hispanics/Latinos, who have one of the highest estimated prevalences of T2DM in the United States [40]. It has been reported that Hispanics with T2DM have more than double the odds of developing dementia than non‐Hispanic patients with T2DM [41]. The adverse health outcomes of T2DM are aggravated in Hispanic individuals by other existing societal disadvantages, such as lack of health insurance coverage [42]. This combination of various sources of disadvantage leads to higher Alzheimer mortality rates related to T2DM. Similarly, Blacks or African Americans are about twice as likely to develop neurodegenerative disorders like AD compared to Whites [43]. These higher rates are compounded by social factors such as limited healthcare access, implicit bias, income inequality and lower health literacy [44, 45]. More attention must be paid to the healthcare requirements of underrepresented populations to identify the multiple factors contributing to health disparities and develop viable solutions at the social, political and medical levels. Given the complex structure of social and biological factors contributing to T2DM, these elements are particularly important in this context [46]. Our study reveals a link between rising T2DM rates and increased Alzheimer's mortality, highlighting the need to identify and address the underlying causes and disparities in T2DM among older adults of distinct ethnicities.

We also observed disparities in Alzheimer's‐related T2DM mortality among individuals living in urban and rural areas, with higher mortality rates in rural areas. Our analysis attests to the greater prevalence of T2DM in rural counties [47]. This also aligns with previous research that linked higher AD‐related mortality to greater physical inactivity, obesity and T2DM [48, 49]. Rural residents often face disadvantages, such as lower healthcare access and socioeconomic status, which may contribute to these disparities [50]. Additionally, the more advanced healthcare infrastructure in urban areas likely supports longer survival with T2DM, in contrast to the less accessible healthcare in rural areas [51, 52]. Educational attainment, one of the most protective factors against Alzheimer's, continues to lag in rural areas relative to urban areas [53]. These reasons tend to explain the disparity between urban and rural areas. Moreover, our state‐wise analysis revealed significant variations in Alzheimer's‐related T2DM mortality across the United States. States with a lower prevalence of T2DM [2] and obesity [54], and higher levels of physical activity [55] tended to exhibit lower Alzheimer's‐related mortality rates among T2DM patients. This underscores the importance of targeted research to elucidate regional disparities and inform state‐specific interventions. Policies promoting active lifestyles and enhancing healthcare facilities are crucial in mitigating the dual burden of T2DM and AD.

AD is a globally prevalent disease that is expected to reach 78 million cases by 2030 [56]. The surge is anticipated to impose substantial medical and economic burdens. Consequently, it is imperative to implement interventions, particularly for T2DM patients, to prevent the development of AD at an early stage. Such measures could significantly reduce both AD incidence and mortality rates in the United States.

### 4.1 Limitations

The CDC WONDER database provided comprehensive access to US mortality records, ensuring a highly representative sample. However, it excludes non‐residents and deaths that occur outside the United States. To remain relevant, we focused on ICD codes specific to T2DM, potentially overlooking broader or unspecified diabetes cases. We recorded the cause of death using ICD‐10 codes to ensure consistent data. However, there is a possibility of coding and diagnostic errors. Our study could not adjust for underlying health conditions or other confounding factors, such as socioeconomic status, type of health insurance, education level and occupation. Additionally, we were unable to include the unreliable data for Hispanics from 1999 in our Joinpoint analysis. The dataset does not permit detailed analysis to explore the root causes of observed trends and disparities. Lastly, this study utilises aggregate data, which may lead to potential biases associated with group‐level inferences, such as ecological bias.

## 5 Conclusion

Our results report a major elevation in Alzheimer's‐related T2DM deaths between 1999 and 2019. Our data also highlight that Hispanic individuals and women are the most impacted by this disparity. The demographic and regional variations demonstrated higher age‐adjusted mortality in rural settings than in urban areas. There is a need for committed strategies to understand and improve the factors that influence disparities in Alzheimer's‐related T2DM mortality among various groups and create interventions that may improve the health outcomes for both T2DM and AD.

## Author Contributions

Saad Ahmed Waqas, Hiba Siddiqui, and Muhammad Umer Sohail conceptualized the manuscript. Taimor Mohammed Khan and Shaheer Qureshi analyzed the data. Dua Ali, Hiba Siddiqui, Maryam Sajid, Zahra Imran, Taimor Mohammed Khan, and Hussain Salim wrote the first draft of the manuscript. Saad Ahmed Waqas and Dua Ali were involved in the process of editing and revising the manuscript. Raheel Ahmed and Shayan Marsia were involved in supervising the project.

## Ethics Statement

The authors have nothing to report.

## Conflicts of Interest

The authors declare no conflicts of interest.

## Supporting information


Data S1.

## Data Availability

The data supporting the findings of this study were obtained from the CDC WONDER online database (Centers for Disease Control and Prevention Wide‐ranging Online Data for Epidemiologic Research). The datasets used and analysed during the current study are publicly available and can be accessed at CDC WONDER (https://wonder.cdc.gov).
