# Supplementary material for: Trends in Alzheimer's‐Related Mortality Among Type 2 Diabetes Patients in the United States: 1999–2019
Source: Endocrinol Diabetes Metab. 2025 Feb 3;8(2):e70032. doi: 10.1002/edm2.70032 (PMC11789764; doi:10.1002/edm2.70032)
Supplement: Supplementary file 1 — Data S1. [file EDM2-8-e70032-s001.docx]

# **Supplementary Material**

| Year | Overall | Male | Female | NH Black or African American | NH White | Hispanic or  Latino | Population |
| --- | --- | --- | --- | --- | --- | --- | --- |
| 1999 | 1408 | 202 | 913 | 129 | 1224 | 38 | 34797841 |
| 2000 | 1793 | 272 | 1186 | 129 | 1577 | 68 | 34991753 |
| 2001 | 1867 | 250 | 1272 | 151 | 1612 | 72 | 35290291 |
| 2002 | 2196 | 319 | 1472 | 167 | 1903 | 88 | 35522207 |
| 2003 | 2385 | 388 | 1539 | 188 | 2058 | 110 | 35863529 |
| 2004 | 2590 | 437 | 1645 | 220 | 2210 | 113 | 36203319 |
| 2005 | 2903 | 514 | 1869 | 254 | 2452 | 141 | 36649798 |
| 2006 | 2922 | 516 | 1892 | 256 | 2468 | 142 | 37164107 |
| 2007 | 3199 | 621 | 2064 | 288 | 2668 | 169 | 37825711 |
| 2008 | 3249 | 683 | 2091 | 282 | 2678 | 207 | 38777621 |
| 2009 | 3306 | 740 | 2082 | 282 | 2714 | 220 | 39623175 |
| 2010 | 3611 | 805 | 2304 | 296 | 2970 | 236 | 40267984 |
| 2011 | 3547 | 756 | 2269 | 316 | 2850 | 258 | 41394141 |
| 2012 | 3532 | 808 | 2292 | 311 | 2832 | 278 | 43145356 |
| 2013 | 3541 | 790 | 2243 | 315 | 2767 | 322 | 44704074 |
| 2014 | 3584 | 877 | 2201 | 314 | 2760 | 361 | 46243211 |
| 2015 | 4049 | 980 | 2543 | 358 | 3050 | 452 | 47760852 |
| 2016 | 4855 | 1207 | 3040 | 459 | 3645 | 524 | 49244195 |
| 2017 | 5389 | 1395 | 3330 | 506 | 4108 | 528 | 50858679 |
| 2018 | 5692 | 1454 | 3485 | 470 | 4262 | 672 | 52431193 |
| 2019 | 5932 | 1580 | 3593 | 520 | 4398 | 712 | 54058263 |
| Total | 71550 | 26225 | 45325 | 6211 | 57206 | 5711 | 8.73E+08 |

**Supplementary Table 1.** Overall, sex-stratified and race-stratified AD and T2DM-related deaths per 100,000 in the United States from 1999 to 2019.

| **Year** | **Medical Facility - Inpatient** | **Medical Facility -Outpatient** | **Decedent’s Home** | **Hospice Facility** | **Nursing Home/Long Term Care Facility** | **Other** | **Place of Death Unknown** |
| --- | --- | --- | --- | --- | --- | --- | --- |
|  |  | **or** |  |  |  |  |  |
|  |  | **ER** |  |  |  |  |  |
| **1999** | 279 | 49 | 147 | Missing | 870 | 50 | Missing |
| **2000** | 339 | 45 | 238 | Missing | 1114 | 45 | Missing |
| **2001** | 312 | 51 | 237 | Missing | 1184 | 61 | Missing |
| **2002** | 379 | 64 | 269 | Missing | 1406 | 63 | Missing |
| **2003** | 400 | 60 | 310 | Missing | 1512 | 85 | Missing |
| **2004** | 404 | 71 | 341 | Missing | 1647 | 115 | Missing |
| **2005** | 449 | 87 | 427 | 23 | 1801 | 103 | Missing |
| **2006** | 397 | 64 | 468 | 35 | 1835 | 98 | 18 |
| **2007** | 426 | 69 | 546 | 37 | 1960 | 136 | 14 |
| **2008** | 469 | 78 | 562 | 51 | 1901 | 147 | 34 |
| **2009** | 405 | 90 | 649 | 77 | 1835 | 182 | 60 |
| **2010** | 415 | 96 | 685 | 76 | 2138 | 195 | Missing |
| **2011** | 387 | 74 | 723 | 96 | 2049 | 212 | Missing |
| **2012** | 308 | 93 | 749 | 80 | 2096 | 199 | Missing |
| **2013** | 346 | 94 | 773 | 106 | 1995 | 214 | Missing |
| **2014** | 306 | 79 | 789 | 104 | 2091 | 208 | Missing |
| **2015** | 336 | 84 | 1003 | 147 | 2216 | 255 | Missing |
| **2016** | 384 | 99 | 1289 | 207 | 2539 | 334 | Missing |
| **2017** | 366 | 122 | 1410 | 199 | 2879 | 411 | Missing |
| **2018** | 421 | 127 | 1602 | 226 | 2946 | 368 | Missing |
| **2019** | 414 | 127 | 1693 | 258 | 3036 | 400 | Missing |
| **Total** | 7942 | 1723 | 14910 | 1722 | 41050 | 3881 | 126 |

**Supplementary Table 2.** AD and T2DM-related mortality per 100,000 stratified by place of death in the United States from 1999 to 2019.

| **Age-Adjusted Rate (95% CI)** | | | |
| --- | --- | --- | --- |
| **Year** | **Female** | **Male** | **Overall** |
| **1999** | 4.08 (3.81-4.35) | 4.08 (3.71-4.44) | 4.12 (3.91-4.34) |
| **2000** | 5.23 (4.94-5.53) | 4.94 (4.54-5.34) | 5.15 (4.91-5.39) |
| **2001** | 5.56 (5.25-5.86) | 4.91 (4.51-5.31) | 5.27 (5.03-5.51) |
| **2002** | 6.38 (6.06-6.71) | 5.81 (5.38-6.24) | 6.15 (5.90-6.41) |
| **2003** | 6.51 (6.19-6.84) | 6.54 (6.09-6.99) | 6.59 (6.33-6.86) |
| **2004** | 6.97 (6.63-7.31) | 7.25 (6.79-7.72) | 7.04 (6.76-7.31) |
| **2005** | 7.76 (7.41-8.12) | 7.71 (7.23-8.18) | 7.75 (7.46-8.03) |
| **2006** | 7.66 (7.31-8.01) | 7.44 (6.98-7.90) | 7.61 (7.34-7.89) |
| **2007** | 8.24 (7.88-8.60) | 7.93 (7.46-8.39) | 8.17 (7.89-8.46) |
| **2008** | 8.21 (7.85-8.56) | 7.92 (7.46-8.38) | 8.15 (7.87-8.43) |
| **2009** | 8.07 (7.72-8.42) | 8.15 (7.69-8.60) | 8.11 (7.83-8.39) |
| **2010** | 8.79 (8.43-9.15) | 8.56 (8.10-9.03) | 8.75 (8.47-9.04) |
| **2011** | 8.51 (8.15-8.87) | 8.04 (7.60-8.49) | 8.35 (8.07-8.63) |
| **2012** | 8.39 (8.04-8.74) | 7.63 (7.20-8.06) | 8.10 (7.83-8.37) |
| **2013** | 8.07 (7.73-8.41) | 7.70 (7.28-8.12) | 7.96 (7.70-8.22) |
| **2014** | 7.74 (7.41-8.07) | 7.98 (7.56-8.40) | 7.87 (7.61-8.13) |
| **2015** | 8.83 (8.48-9.18) | 8.43 (8.01-8.86) | 8.71 (8.44-8.98) |
| **2016** | 10.35 (9.98-10.73) | 9.91 (9.45-10.37) | 10.18 (9.89-10.47) |
| **2017** | 11.08 (10.70-11.46) | 10.90 (10.42-11.37) | 11.03 (10.74-11.33) |
| **2018** | 11.51 (11.12-11.89) | 11.32 (10.85-11.80) | 11.41 (11.11-11.71) |
| **2019** | 11.61 (11.23-11.99) | 11.70 (11.22-12.17) | 11.65 (11.36-11.95) |
| **Total** | 8.25 (8.17-8.33) | 8.19 (8.09-8.29) | 8.23 (8.17-8.29) |

**Supplementary Table 3.** Overall and sex-stratified AD and T2DM-related AAMR per 100,000 in the United States from 1999 to 2019.

| Crude Rate per 100,000 (95% CI) | | | |
| --- | --- | --- | --- |
| Year | **65-74** | **75-84** | **85+** |
| 1999 | 0.58 (0.47-0.69) | 5.10 (4.70-5.51) | 16.30 (15.07-17.53) |
| 2000 | 0.81 (0.68-0.94) | 6.12 (5.68-6.55) | 20.95 (19.57-22.32) |
| 2001 | 0.73 (0.61-0.86) | 6.03 (5.60-6.46) | 22.56 (21.15-23.98) |
| 2002 | 1.09 (0.94-1.24) | 6.89 (6.44-7.35) | 25.55 (24.05-27.04) |
| 2003 | 0.85 (0.72-0.99) | 7.60 (7.12-8.08) | 27.92 (26.37-29.47) |
| 2004 | 0.92 (0.78-1.05) | 8.31 (7.82-8.81) | 29.46 (27.88-31.03) |
| 2005 | 1.12 (0.97-1.27) | 8.86 (8.35-9.37) | 32.69 (31.05-34.32) |
| 2006 | 1.03 (0.89-1.18) | 8.58 (8.08-9.09) | 32.88 (31.27-34.49) |
| 2007 | 1.17 (1.02-1.32) | 9.34 (8.81-9.86) | 34.65 (33.02-36.27) |
| 2008 | 0.96 (0.82-1.09) | 9.42 (8.90-9.95) | 35.05 (33.44-36.66) |
| 2009 | 1.10 (0.96-1.24) | 9.23 (8.71-9.75) | 34.84 (33.26-36.42) |
| 2010 | 1.08 (0.94-1.22) | 9.76 (9.23-10.30) | 38.26 (36.63-39.90) |
| 2011 | 1.08 (0.95-1.22) | 9.47 (8.94-9.99) | 35.85 (34.30-37.40) |
| 2012 | 0.91 (0.79-1.03) | 9.44 (8.92-9.96) | 34.99 (33.48-36.50) |
| 2013 | 0.98 (0.86-1.10) | 9.10 (8.59-9.61) | 34.27 (32.79-35.74) |
| 2014 | 0.99 (0.87-1.11) | 8.67 (8.18-9.16) | 34.70 (33.23-36.17) |
| 2015 | 1.03 (0.91-1.15) | 9.85 (9.33-10.38) | 38.06 (36.54-39.59) |
| 2016 | 1.15 (1.02-1.27) | 10.97 (10.43-11.52) | 46.47 (44.80-48.14) |
| 2017 | 1.41 (1.27-1.54) | 11.63 (11.08-12.19) | 50.41 (48.68-52.14) |
| 2018 | 1.32 (1.19-1.45) | 12.29 (11.74-12.84) | 51.91 (50.16-53.65) |
| 2019 | 1.34 (1.22-1.47) | 12.58 (12.03-13.13) | 52.99 (51.24-54.75) |
| Total | 1.06 (1.03-1.09) | 9.13 (9.02-9.24) | 36.11 (35.76-36.46) |

**Supplementary Table 4.** AD and T2DM-related crude mortality rate per 100,000 stratified by age group in the United States from 1999 to 2019.

| Year | NH Black or African American | NH White | Hispanic or Latino |
| --- | --- | --- | --- |
| 1999 | 5.03 (4.16-5.90) | 4.14 (3.90-4.37) | 2.90 (2.04-4.00) |
| 2000 | 4.93 (4.07-5.78) | 5.22 (4.96-5.48) | 4.99 (3.87-6.33) |
| 2001 | 5.74 (4.82-6.66) | 5.28 (5.02-5.54) | 4.94 (3.85-6.24) |
| 2002 | 6.28 (5.32-7.23) | 6.18 (5.91-6.46) | 5.65 (4.52-6.98) |
| 2003 | 7.06 (6.05-8.08) | 6.61 (6.33-6.90) | 6.78 (5.49-8.06) |
| 2004 | 8.18 (7.09-9.26) | 7.07 (6.78-7.37) | 6.60 (5.36-7.83) |
| 2005 | 9.29 (8.15-10.43) | 7.70 (7.39-8.00) | 7.61 (6.34-8.88) |
| 2006 | 9.16 (8.04-10.29) | 7.56 (7.26-7.86) | 7.47 (6.23-8.71) |
| 2007 | 10.08 (8.91-11.24) | 8.11 (7.80-8.42) | 8.46 (7.18-9.75) |
| 2008 | 9.65 (8.53-10.78) | 7.96 (7.65-8.26) | 9.69 (8.36-11.02) |
| 2009 | 9.30 (8.21-10.38) | 7.95 (7.65-8.25) | 9.77 (8.47-11.06) |
| 2010 | 9.54 (8.45-10.63) | 8.56 (8.25-8.87) | 9.98 (8.71-11.26) |
| 2011 | 9.91 (8.81-10.99) | 8.07 (7.78-8.37) | 10.06 (8.83-11.29) |
| 2012 | 9.31 (8.27-10.35) | 7.88 (7.59-8.17) | 10.20 (9.00-11.40) |
| 2013 | 9.09 (8.08-10.10) | 7.60 (7.31-7.88) | 11.09 (9.88-12.31) |
| 2014 | 8.80 (7.82-9.78) | 7.45 (7.17-7.73) | 11.52 (10.32-12.71) |
| 2015 | 9.64 (8.64-10.65) | 8.15 (7.86-8.44) | 13.47 (12.22-14.71) |
| 2016 | 12.08 (10.97-13.19) | 9.54 (9.23-9.86) | 15.03 (13.74-16.32) |
| 2017 | 12.80 (11.67-13.92) | 10.59 (10.27-10.92) | 14.14 (12.93-15.35) |
| 2018 | 11.47 (10.43-12.51) | 10.83 (10.50-11.16) | 17.17 (15.87-18.48) |
| 2019 | 12.32 (11.26-13.39) | 11.01 (10.68-11.33) | 17.44 (16.15-18.73) |
| Total | 9.30 (9.06-9.53) | 7.92 (7.85-7.98) | 17.44 (16.15-18.73) |

**Supplementary Table 5**. AD and T2DM-related AAMR per 100,000 stratified by race in the United States from 1999 to 2019.

| Age-Adjusted Rate (95% CI) | | | |
| --- | --- | --- | --- |
| Year | **Large Metropolitan** | **Medium/Small Metropolitan** | **Non-Metropolitan** |
| 1999 | 3.10 (2.84-3.36) | 4.89 (4.47-5.31) | 5.42 (4.86-5.97) |
| 2000 | 3.84 (3.55-4.13) | 6.06 (5.59-6.52) | 7.06 (6.42-7.69) |
| 2001 | 3.97 (3.68-4.27) | 6.13 (5.66-6.60) | 7.39 (6.74-8.03) |
| 2002 | 4.55 (4.24-4.87) | 7.11 (6.61-7.61) | 8.88 (8.18-9.59) |
| 2003 | 4.63 (4.31-4.94) | 7.96 (7.44-8.49) | 9.45 (8.73-10.18) |
| 2004 | 5.17 (4.84-5.49) | 8.19 (7.66-8.71) | 10.07 (9.32-10.82) |
| 2005 | 5.70 (5.36-6.04) | 9.13 (8.58-9.68) | 10.89 (10.11-11.66) |
| 2006 | 5.79 (5.45-6.13) | 8.60 (8.08-9.13) | 10.89 (10.12-11.66) |
| 2007 | 6.11 (5.77-6.46) | 9.18 (8.64-9.72) | 12.10 (11.29-12.90) |
| 2008 | 6.29 (5.95-6.64) | 9.12 (8.59-9.65) | 11.55 (10.77-12.34) |
| 2009 | 6.43 (6.08-6.77) | 9.28 (8.75-9.81) | 10.84 (10.08-11.59) |
| 2010 | 6.76 (6.41-7.12) | 10.04 (9.49-10.59) | 11.96 (11.17-12.74) |
| 2011 | 6.66 (6.31-7.00) | 9.28 (8.76-9.80) | 11.40 (10.63-12.16) |
| 2012 | 6.77 (6.42-7.11) | 8.73 (8.23-9.23) | 10.91 (10.17-11.66) |
| 2013 | 6.93 (6.59-7.28) | 8.35 (7.87-8.83) | 10.08 (9.37-10.79) |
| 2014 | 6.55 (6.22-6.88) | 8.77 (8.28-9.25) | 9.87 (9.17-10.56) |
| 2015 | 7.69 (7.33-8.04) | 9.36 (8.86-9.86) | 10.57 (9.85-11.28) |
| 2016 | 8.70 (8.33-9.08) | 11.35 (10.81-11.89) | 12.55 (11.78-13.33) |
| 2017 | 9.44 (9.06-9.82) | 11.94 (11.40-12.49) | 14.17 (13.35-14.98) |
| 2018 | 9.83 (9.45-10.22) | 12.90 (12.34-13.46) | 13.33 (12.55-14.11) |
| 2019 | 10.15 (9.76-10.53) | 12.70 (12.14-13.25) | 14.31 (13.50-15.11) |
| Total | 6.68 (6.60-6.76) | 9.25 (9.14-9.37) | 10.74 (10.58-10.90) |

**Supplementary Table 6.** AD and T2DM-related AAMR per 100,000 stratified by Urban-Rural classification in the United States from 1999 to 2019.

| **Census Region** | **Year** | | **Age Adjusted Mortality Rate** |
| --- | --- | --- | --- |
| **Census Region 1: Northeast** | **1999** | | 3.13 (2.73-3.53) |
| **Census Region 1: Northeast** | **2000** | | 3.97 (3.52-4.42) |
| **Census Region 1: Northeast** | **2001** | | 3.74 (3.31-4.18) |
| **Census Region 1: Northeast** | **2002** | | 4.42 (3.95-4.89) |
| **Census Region 1: Northeast** | **2003** | | 4.34 (3.88-4.80) |
| **Census Region 1: Northeast** | **2004** | | 4.90 (4.42-5.39) |
| **Census Region 1: Northeast** | **2005** | | 5.05 (4.56-5.54) |
| **Census Region 1: Northeast** | **2006** | | 5.13 (4.64-5.62) |
| **Census Region 1: Northeast** | **2007** | | 4.95 (4.46-5.43) |
| **Census Region 1: Northeast** | **2008** | | 4.66 (4.20-5.13) |
| **Census Region 1: Northeast** | **2009** | | 4.62 (4.17-5.08) |
| **Census Region 1: Northeast** | **2010** | | 4.75 (4.29-5.21) |
| **Census Region 1: Northeast** | **2011** | | 4.26 (3.83-4.69) |
| **Census Region 1: Northeast** | **2012** | | 4.56 (4.11-5.01) |
| **Census Region 1: Northeast** | **2013** | | 4.03 (3.61-4.45) |
| **Census Region 1: Northeast** | **2014** | | 3.98 (3.57-4.40) |
| **Census Region 1: Northeast** | **2015** | | 4.37 (3.94-4.81) |
| **Census Region 1: Northeast** | **2016** | | 5.00 (4.54-5.45) |
| **Census Region 1: Northeast** | **2017** | | 5.47 (4.99-5.94) |
| **Census Region 1: Northeast** | **2018** | | 6.18 (5.68-6.68) |
| **Census Region 1: Northeast** | **2019** | | 6.04 (5.56-6.53) |
| **Total** | | 4.70 (4.60-4.80) | |
| **Census Region 2: Midwest** | **1999** | | 4.97 (4.49-5.44) |
| **Census Region 2: Midwest** | **2000** | | 6.47 (5.93-7.02) |
| **Census Region 2: Midwest** | **2001** | | 6.66 (6.11-7.20) |
| **Census Region 2: Midwest** | **2002** | | 7.93 (7.34-8.53) |
| **Census Region 2: Midwest** | **2003** | | 8.64 (8.02-9.25) |
| **Census Region 2: Midwest** | **2004** | | 8.81 (8.19-9.42) |
| **Census Region 2: Midwest** | **2005** | | 9.98 (9.33-10.63) |
| **Census Region 2: Midwest** | **2006** | | 8.83 (8.22-9.44) |
| **Census Region 2: Midwest** | **2007** | | 9.83 (9.19-10.47) |
| **Census Region 2: Midwest** | **2008** | | 9.92 (9.28-10.56) |
| **Census Region 2: Midwest** | **2009** | | 9.41 (8.79-10.02) |
| **Census Region 2: Midwest** | **2010** | | 10.27 (9.63-10.91) |
| **Census Region 2: Midwest** | **2011** | | 9.78 (9.16-10.41) |
| **Census Region 2: Midwest** | **2012** | | 9.76 (9.14-10.37) |
| **Census Region 2: Midwest** | **2013** | | 9.00 (8.41-9.59) |
| **Census Region 2: Midwest** | **2014** | | 8.93 (8.34-9.51) |
| **Census Region 2: Midwest** | **2015** | | 9.45 (8.85-10.04) |
| **Census Region 2: Midwest** | **2016** | | 11.80 (11.14-12.46) |
| **Census Region 2: Midwest** | **2017** | | 12.75 (12.08-13.43) |
| **Census Region 2: Midwest** | **2018** | | 12.60 (11.93-13.27) |
| **Census Region 2: Midwest** | **2019** | | 13.35 (12.67-14.04) |
| **Total** | | 9.62 (9.49-9.76) | |
| **Census Region 3: South** | **1999** | | 4.58 (4.19-4.97) |
| **Census Region 3: South** | **2000** | | 5.43 (5.01-5.85) |
| **Census Region 3: South** | **2001** | | 5.52 (5.10-5.94) |
| **Census Region 3: South** | **2002** | | 6.15 (5.71-6.59) |
| **Census Region 3: South** | **2003** | | 6.83 (6.37-7.30) |
| **Census Region 3: South** | **2004** | | 7.15 (6.68-7.62) |
| **Census Region 3: South** | **2005** | | 7.97 (7.48-8.46) |
| **Census Region 3: South** | **2006** | | 7.81 (7.33-8.28) |
| **Census Region 3: South** | **2007** | | 8.38 (7.89-8.86) |
| **Census Region 3: South** | **2008** | | 7.81 (7.35-8.28) |
| **Census Region 3: South** | **2009** | | 8.20 (7.73-8.68) |
| **Census Region 3: South** | **2010** | | 8.54 (8.06-9.02) |
| **Census Region 3: South** | **2011** | | 8.17 (7.71-8.63) |
| **Census Region 3: South** | **2012** | | 7.42 (6.99-7.86) |
| **Census Region 3: South** | **2013** | | 6.77 (6.36-7.17) |
| **Census Region 3: South** | **2014** | | 6.87 (6.46-7.27) |
| **Census Region 3: South** | **2015** | | 7.57 (7.15-7.99) |
| **Census Region 3: South** | **2016** | | 8.69 (8.25-9.14) |
| **Census Region 3: South** | **2017** | | 9.79 (9.32-10.25) |
| **Census Region 3: South** | **2018** | | 10.06 (9.60-10.52) |
| **Census Region 3: South** | **2019** | | 10.10 (9.64-10.55) |
| **Total** | | 7.77 (7.67-7.87) | |
| **Census Region 4: West** | **1999** | | 3.29 (2.85-3.73) |
| **Census Region 4: West** | **2000** | | 4.42 (3.91-4.92) |
| **Census Region 4: West** | **2001** | | 4.97 (4.45-5.50) |
| **Census Region 4: West** | **2002** | | 5.83 (5.27-6.40) |
| **Census Region 4: West** | **2003** | | 6.07 (5.50-6.63) |
| **Census Region 4: West** | **2004** | | 6.92 (6.32-7.52) |
| **Census Region 4: West** | **2005** | | 7.51 (6.90-8.13) |
| **Census Region 4: West** | **2006** | | 8.41 (7.77-9.05) |
| **Census Region 4: West** | **2007** | | 9.27 (8.60-9.93) |
| **Census Region 4: West** | **2008** | | 10.19 (9.50-10.88) |
| **Census Region 4: West** | **2009** | | 9.98 (9.31-10.66) |
| **Census Region 4: West** | **2010** | | 11.24 (10.53-11.95) |
| **Census Region 4: West** | **2011** | | 10.94 (10.26-11.63) |
| **Census Region 4: West** | **2012** | | 10.88 (10.20-11.55) |
| **Census Region 4: West** | **2013** | | 12.47 (11.75-13.19) |
| **Census Region 4: West** | **2014** | | 11.91 (11.22-12.59) |
| **Census Region 4: West** | **2015** | | 13.76 (13.03-14.49) |
| **Census Region 4: West** | **2016** | | 15.65 (14.89-16.42) |
| **Census Region 4: West** | **2017** | | 16.47 (15.69-17.25) |
| **Census Region 4: West** | **2018** | | 17.09 (16.32-17.87) |
| **Census Region 4: West** | **2019** | | 17.43 (16.66-18.21) |
| **Total** | | 10.91 (10.75-11.06) | |

**Supplementary Table 7.** AD and T2DM-related AAMR per 100,000 stratified by census region in the United States from 1999 to 2019.

| **State** | **Age-Adjusted Rate (95% CI)** |
| --- | --- |
| **North Dakota** | 16.03 (14.44 - 17.62) |
| **Washington** | 15.00 (14.43 - 15.57) |
| **Tennessee** | 14.25 (13.67 - 14.82) |
| **West Virginia** | 13.59 (12.66 - 14.52) |
| **Kentucky** | 13.04 (12.38 - 13.70) |
| **South Dakota** | 12.90 (11.57 - 14.23) |
| **California** | 12.61 (12.38 - 12.84) |
| **Iowa** | 12.43 (11.77 - 13.10) |
| **Nebraska** | 12.24 (11.33 - 13.14) |
| **Ohio** | 12.00 (11.64 - 12.36) |
| **Minnesota** | 11.95 (11.41 - 12.49) |
| **Vermont** | 11.74 (10.23 - 13.26) |
| **Indiana** | 11.71 (11.21 - 12.21) |
| **Texas** | 11.60 (11.30 - 11.89) |
| **Oregon** | 11.54 (10.92 - 12.16) |
| **Wyoming** | 10.63 (8.93 - 12.33) |
| **Oklahoma** | 10.58 (9.95 - 11.20) |
| **Idaho** | 10.14 (9.15 - 11.14) |
| **South Carolina** | 10.12 (9.55 - 10.69) |
| **Alabama** | 9.41 (8.88 - 9.94) |
| **Wisconsin** | 9.38 (8.93 - 9.83) |
| **Colorado** | 9.32 (8.75 - 9.89) |
| **North Carolina** | 9.24 (8.85 - 9.62) |
| **Montana** | 9.10 (8.05 - 10.16) |
| **Kansas** | 8.05 (7.46 - 8.65) |
| **Missouri** | 7.93 (7.51 - 8.34) |
| **Michigan** | 7.90 (7.58 - 8.22) |
| **Maine** | 7.58 (6.77 - 8.38) |
| **Pennsylvania** | 7.31 (7.06 - 7.56) |
| **Delaware** | 7.06 (6.03 - 8.08) |
| **New Hampshire** | 6.83 (6.00 - 7.65) |
| **Rhode Island** | 6.60 (5.77 - 7.43) |
| **Mississippi** | 6.55 (5.98 - 7.13) |
| **Alaska** | 6.33 (4.77 - 8.24) |
| **Utah** | 6.16 (5.48 - 6.85) |
| **Illinois** | 6.04 (5.79 - 6.30) |
| **Hawaii** | 6.01 (5.29 - 6.72) |
| **Georgia** | 5.80 (5.46 - 6.14) |
| **Virginia** | 5.77 (5.43 - 6.10) |
| **New Mexico** | 5.66 (5.02 - 6.30) |
| **Arizona** | 5.49 (5.14 - 5.83) |
| **Maryland** | 5.32 (4.95 - 5.69) |
| **Arkansas** | 5.20 (4.72 - 5.68) |
| **Connecticut** | 4.39 (4.01 - 4.77) |
| **District of Columbia** | 4.34 (3.37 - 5.49) |
| **New Jersey** | 4.18 (3.94 - 4.43) |
| **Louisiana** | 3.72 (3.37 - 4.08) |
| **Florida** | 3.39 (3.26 - 3.53) |
| **Massachusetts** | 3.35 (3.10 - 3.59) |
| **Nevada** | 3.09 (2.62 - 3.55) |

**Supplementary Table 8.** AD and T2DM-related AAMR per 100,000 stratified by state in the United States from 1999 to 2019.


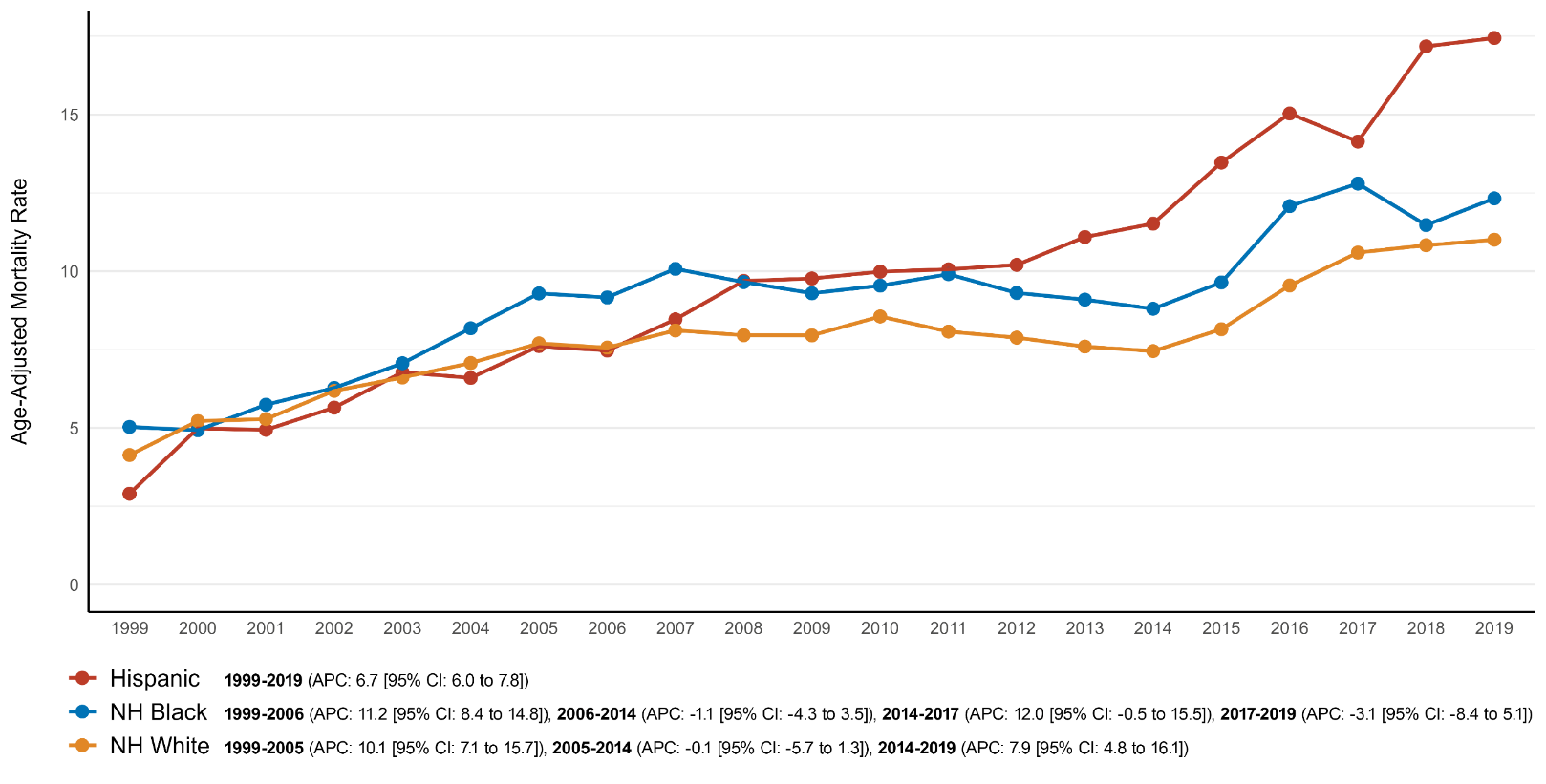


**Supplementary Figure 1.** Trends in AD and T2DM-related AAMR per 100,000 stratified by race in the United States from 1999 to 2019.


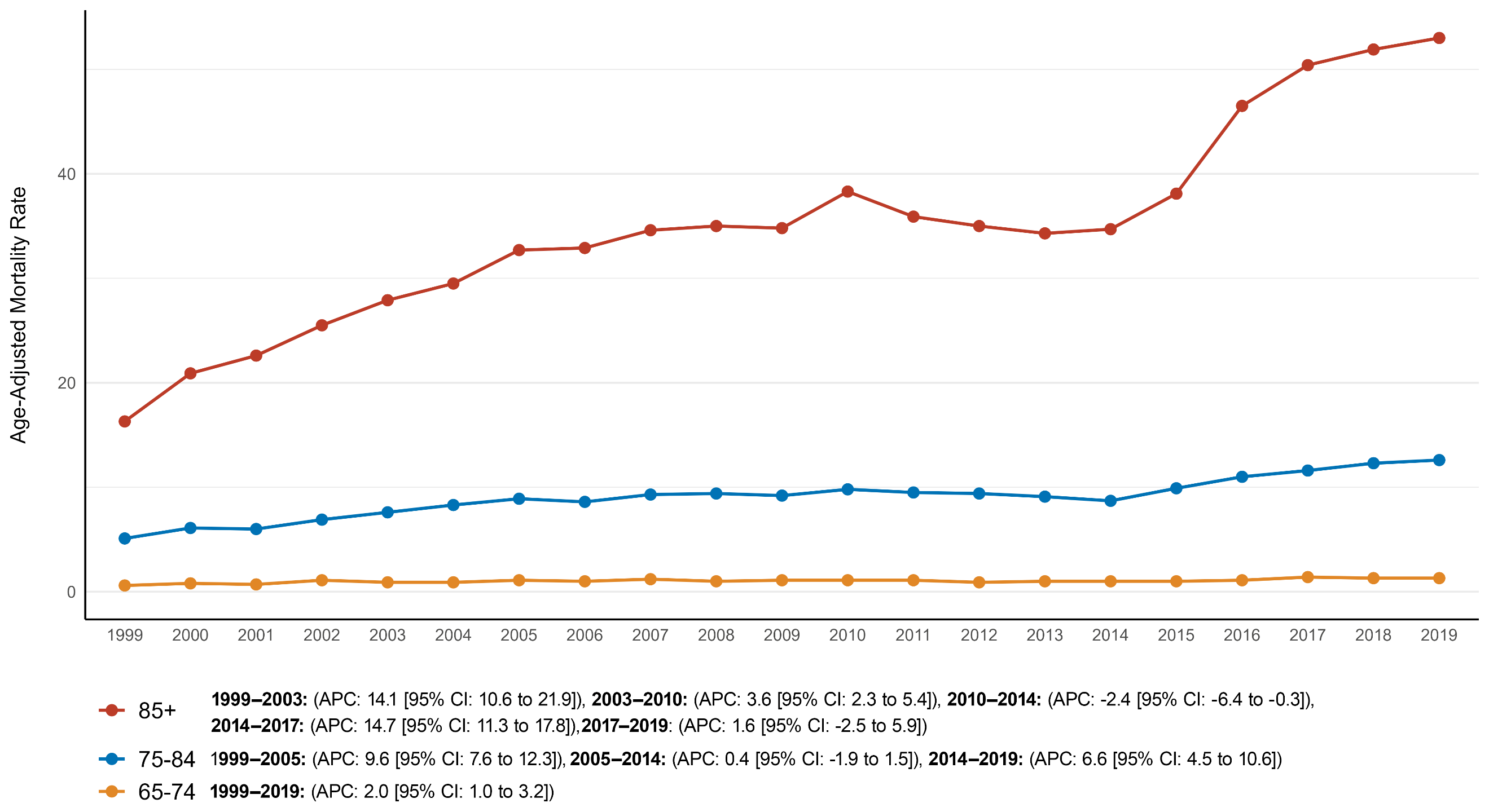


**Supplementary Figure 2.** Trends in AD and T2DM-related AAMR per 100,000 stratified by 10-year age groups in the United States from 1999 to 2019.


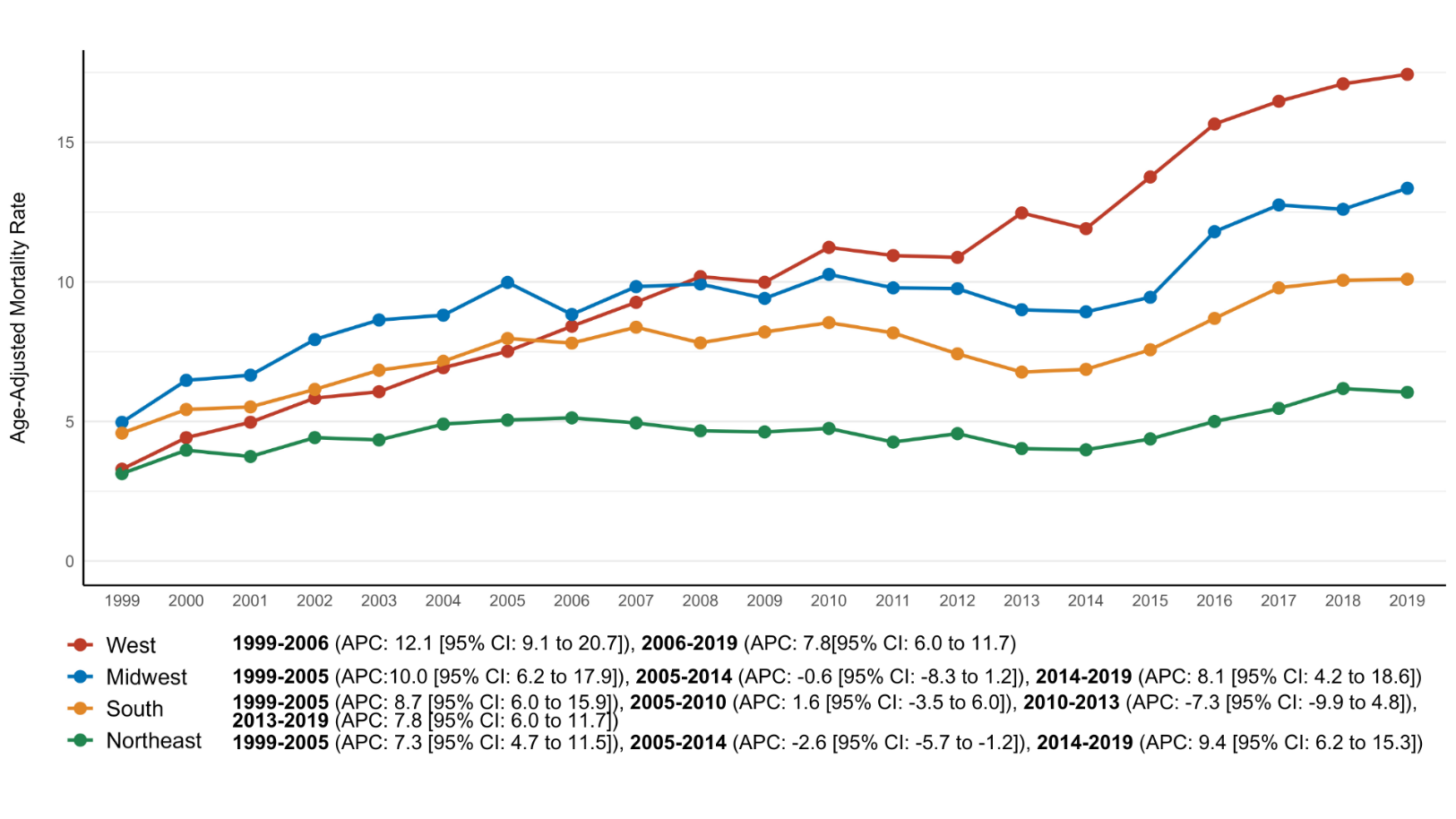


**Supplementary Figure 3.** Trends in AD and T2DM-related AAMR per 100,000 stratified by region in the United States from 1999 to 2019.


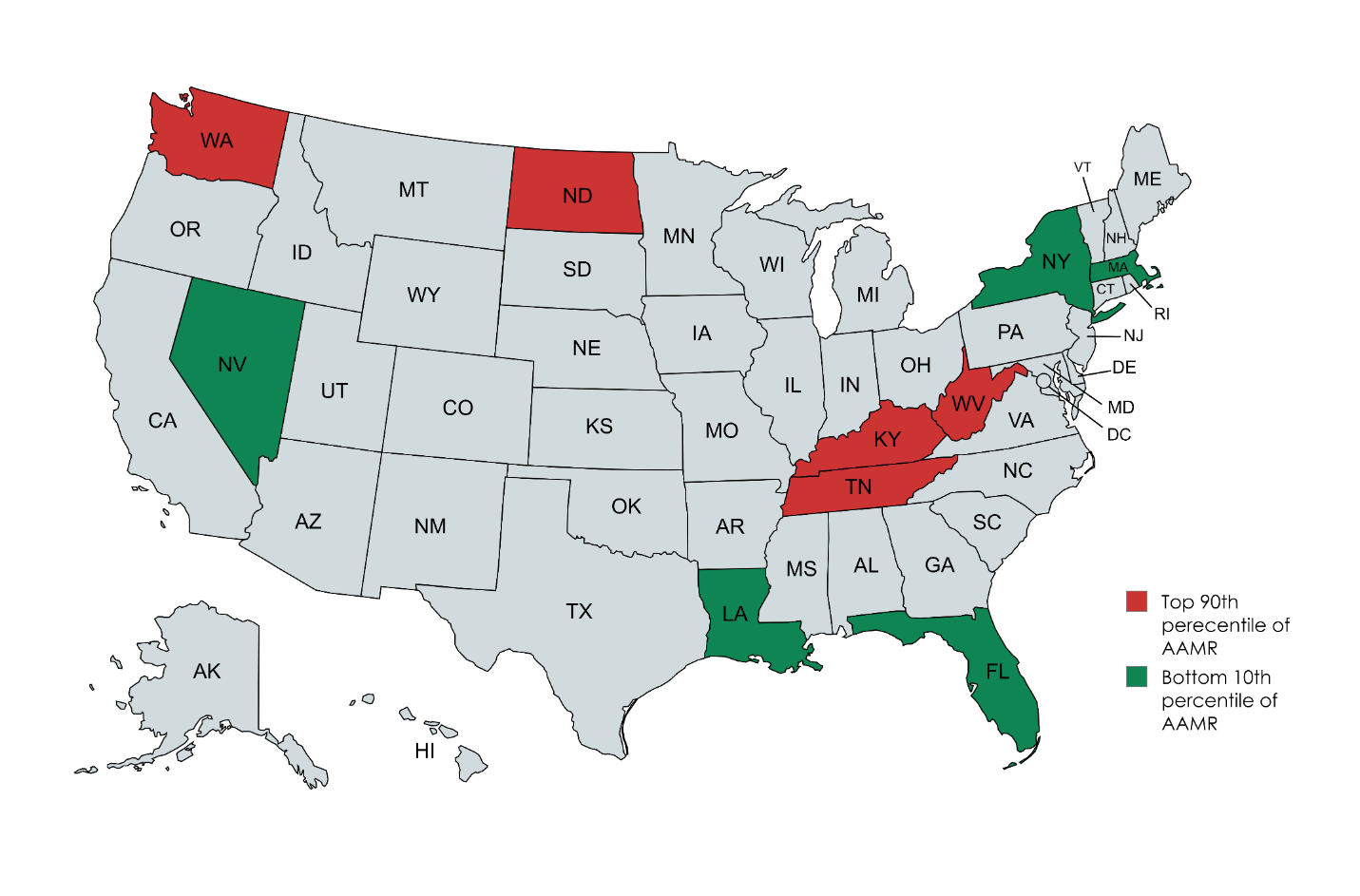


**Supplementary Figure 4.** AD and T2DM-related AAMR per 100,000 stratified by states in top 90^th^ percentile and bottom 10^th^ percentile in the United States from 1999 to 2019.
